# Choriocarcinoma brain metastasis in a patient in the third trimester: a case report

**DOI:** 10.1186/s13256-021-02808-3

**Published:** 2021-07-29

**Authors:** Chunjuan Shen, Ling Ai, Kai Li, Yunfei Cao, Hanbing Wu, Dandan Sun

**Affiliations:** 1 Department of Obstetrics and Gynecology, Jiaxing Maternity and Child Health Care Hospital, Jiaxing University Affiliated Maternity and Child Hospital, Jiaxing, Zhejiang China; 2 Department of Orthopedics, The Second Hospital of Jiaxing, Jiaxing, Zhejiang China

**Keywords:** Choriocarcinoma, Pregnancy, Metastasis, Chemotherapy, Elective caesarean section

## Abstract

**Background:**

Metastatic choriocarcinoma in the third trimester of pregnancy is extremely rare.

**Case presentation:**

A 25-year-old Chinese woman (gravida 3, para 0) who was 28 weeks pregnant was admitted for sudden convulsion, aconuresis, and unconsciousness. The decision was made to perform an emergency cesarean delivery and craniotomy, hematoma clearance, and decompression. Pathological examination confirmed choriocarcinoma with brain metastasis. The patient underwent chemotherapy with the etoposide, cisplatin (EP) and etoposide, methotrexate and dactinomycin alternating with cyclophosphamide and vincristine (EMACO) regimens. A satisfactory result was achieved.

**Conclusions:**

When encountering intracranial mass or bilateral pulmonary nodules in a pregnant woman, especially one in the third trimester, metastatic choriocarcinoma should be considered.

## Introduction

Choriocarcinoma may develop during any form of gestation: abortion or tubal pregnancy, term or preterm gestation, or hydatidiform moles. Clinical follow-up is considered to be essential due to the high risk of metastases. Outside the pelvis, lung metastases are relatively common, occurring in 80% of cases; brain metastases are relatively uncommon (10%) [1]. Here we report a rare case of a young pregnant woman (gravida 3, para 0) with choriocarcinoma brain metastasis in the 28th gestational week. The aim of this report was to present this rare case and discuss the management of choriocarcinoma with brain metastasis.

## Case report

A 25-year-old Chinese woman in her 28th week of pregnancy was admitted to the local hospital with a complaint of severe and unremitting headaches, nausea, and vomiting. The patient was given metoclopramide (dose 10 mg) by intramuscular injection to alleviate her symptoms, with no effect. She was then transferred to Jiaxing Maternity and Child Health Care Hospital. After admission, she presented sudden convulsion, aconuresis, and unconsciousness. Blood pressure, pulse, and temperature were 90–100/60–70 mmHg, 90–100 bpm, and 36.7–37 ℃, respectively. The patient was administered intravenous mannitol (1 g/kg over a 15-min period).

The patient’s medical history revealed that her last pregnancy was terminated by artificial abortion in August 2017. There had also been one previous artificial abortion 3 years earlier. After the last artificial abortion, the patient's menstrual cycle was regular, and there was no irregular uterine bleeding. The patient had no other special family, medical, and surgical history. After leaving college, she worked as a secretary and she was registered as a resident of Jiaxing Zhejiang. The patient had never smoked or consumed alcohol.

### Examination

Blood pressure, pulse, and temperature were 90/58 mmHg, 110 bpm, and 36.3 ℃, respectively. On examination, she had no signs of brain trauma. Meningeal signs were positive, with cervical rigidity. Coma was absent; there was a minimally reactive 6-mm right pupil with pupillary reaction to light and a reactive 5-mm left pupil with a faint pupillary reaction to light. In muscle group I the muscular tension in the limbs was increased. The Glascow Coma Scale score was 5. She had normal heart sounds and normal breath sounds of the lungs. The abdominal examination revealed central obesity and a bulky uterus with a size corresponding to 28 weeks of gestation. No adnexal masses were noted. Computerized tomography (CT) scanning of the brain revealed a hemorrhagic mass in the right occipital region extending into the ventricle system, with herniation of the brain. Uterine ultrasonographic depicted 28-week-old fetus and the absence of any abnormal mass.

### Operation

**Fig. 1:**
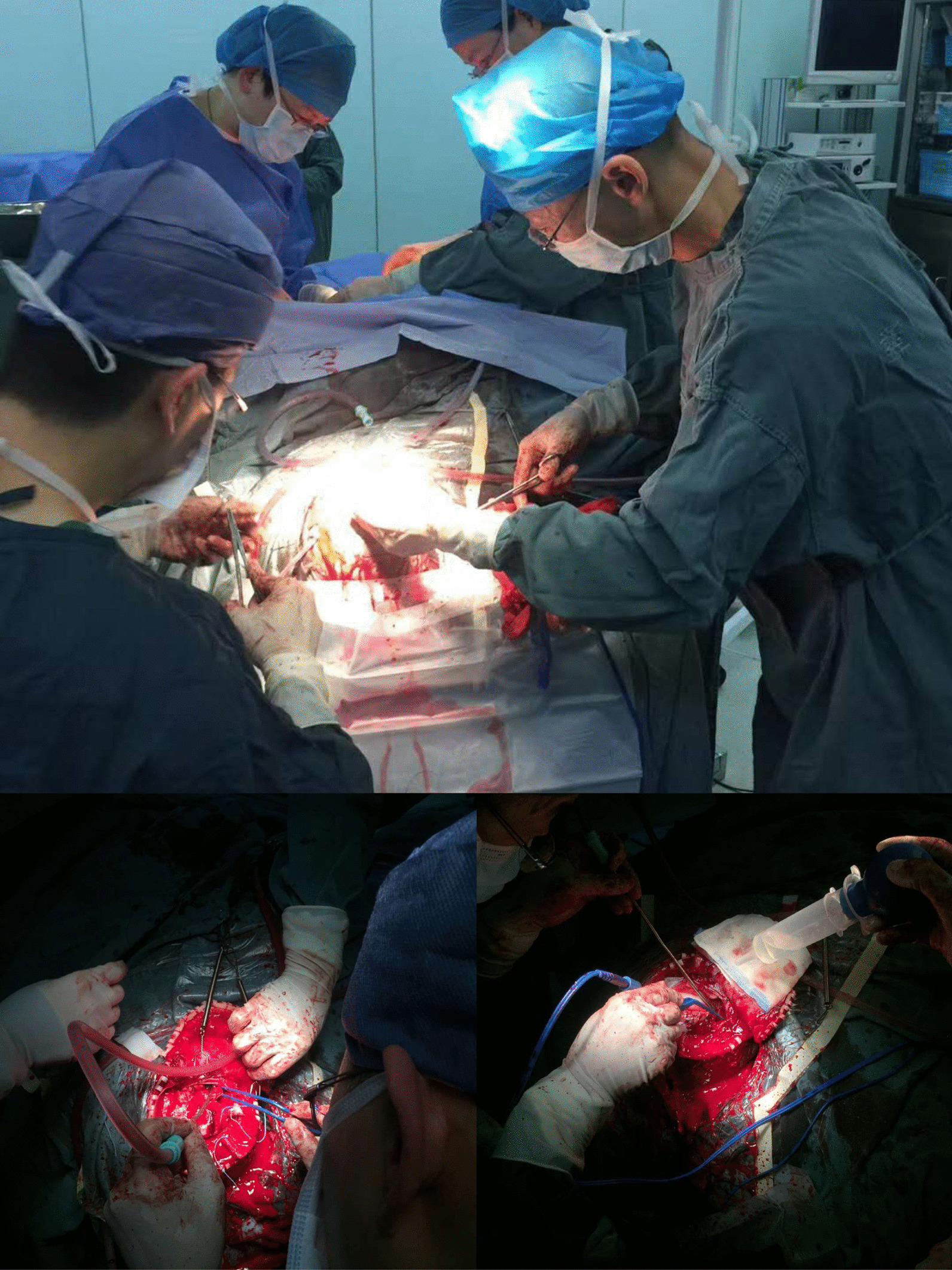
Intraoperative picture

A tentative plan was made for an emergency cesarean delivery and craniotomy, hematoma clearance, and decompression (Fig. 1). The fetus was delivered, with Apgar scores at 1.5 min of 2 and 3 and a body weight of 1180 g, and the newborn was admitted to the neonatal intensive care unit. The uterus, pelvis, and abdomen showed no definitive abnormalities. Areas of focal hemorrhage were removed and brain pressure relaxed; all vital signs were within normal limits.

### Postoperative

**Fig. 2:**
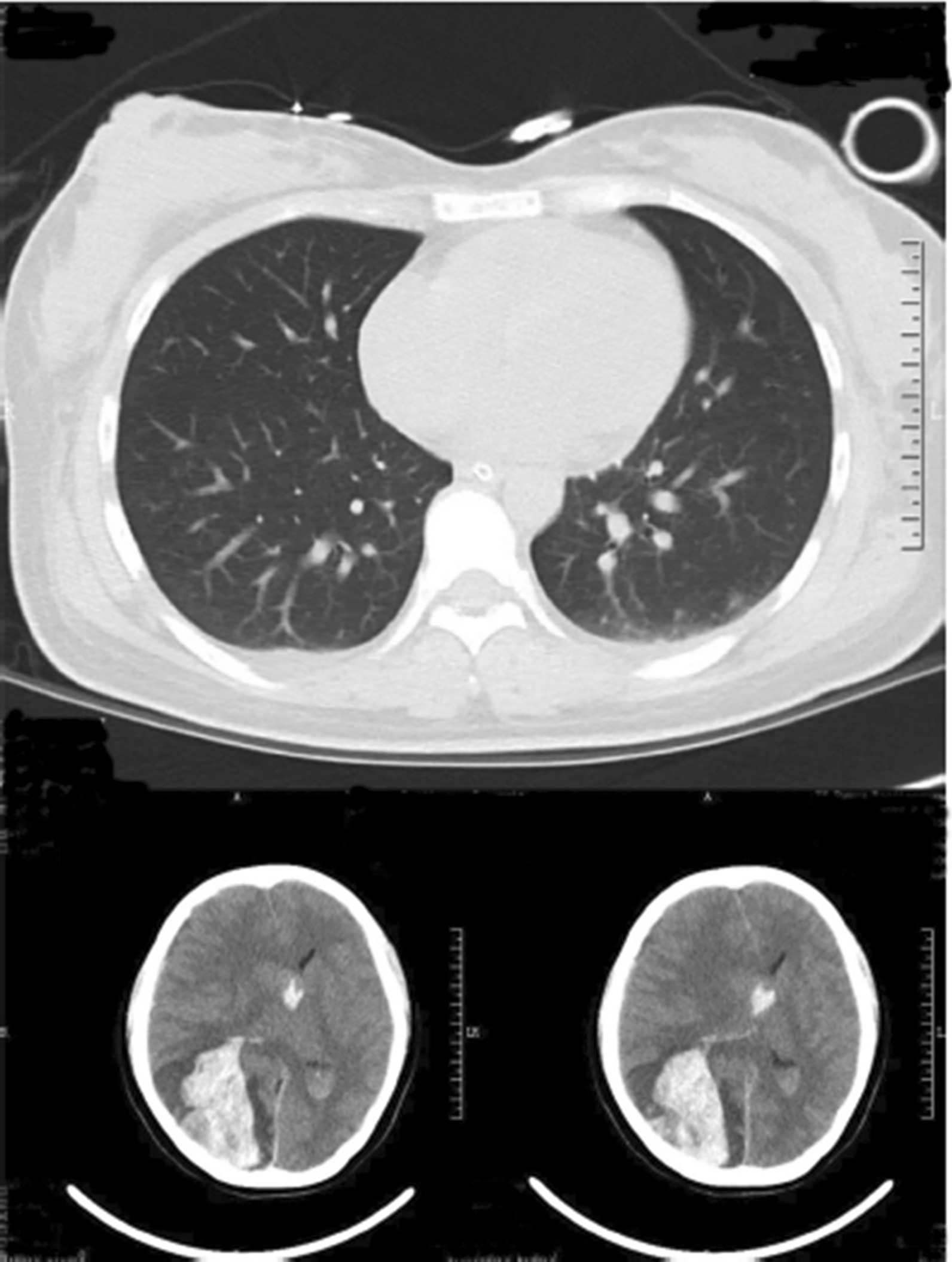
Computerized tomography scann of the lung and brain

The patient recovered consciousness, and the neurological symptoms were relieved at 1 day after the surgery. Re-examination of the CT scan of the brain showed that the right occipital hematoma was essentially removed, and CT scan of the lung showed a mass in the upper lobe of the right lung consisting of irregular, multiple bilateral pulmonary nodules (Fig. 2).

### Pathological findings

**Fig. 3:**
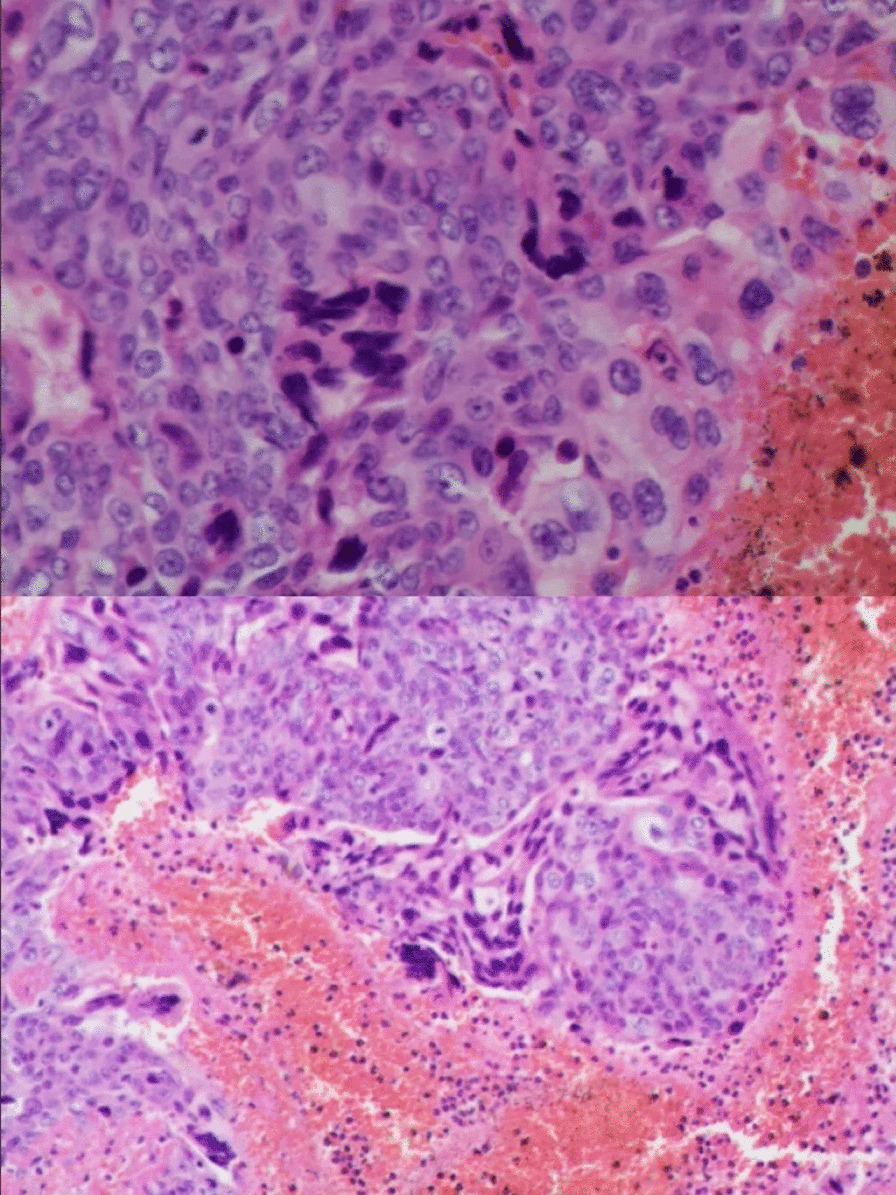
Pathological examination of the brain hemorrhagic mass

Pathological examination of the brain hemorrhagic mass showed a histological pattern and structure of metastatic choriocarcinoma. Immunohistochemistry was: CK(pan)(+), HCG(+), and HPL(+) (Fig. 3).

### Postoperative therapy

**Fig. 4:**
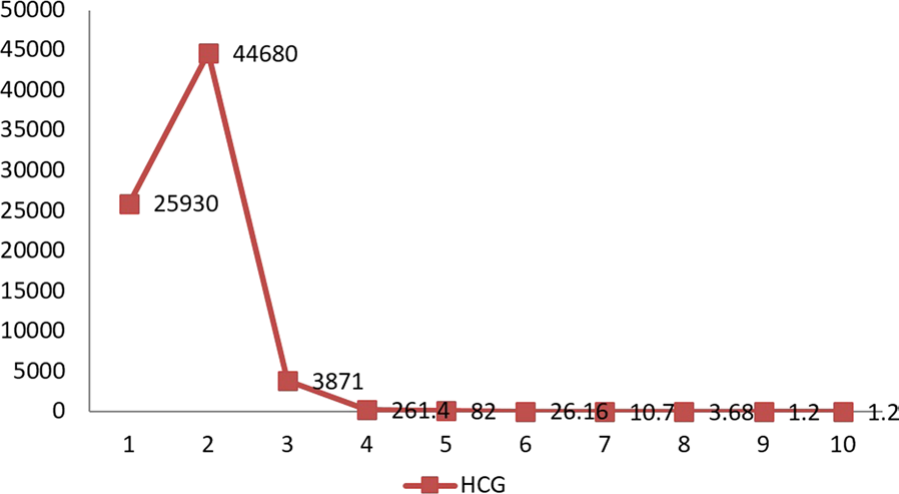
The level of serum beta-human chorionic gonadotropin (*HCG*) after chemotherapy.

The patient began chemotherapy at 7 days after the surgery with a regimen of etoposide and cisplatin (EP) in the first two courses of treatment (etoposide 100 mg/m^2^,* cis*-platinum 20 mg/m^2^). She received an injection of palonosetron hydrochloride (dose 0.25 mg) intravenously about 30 min before each chemotherapy course. The duration of one course was 2 days, and the interval between each course was 1 week. She was started on the EMACO regimen as the third course of treatment (etoposide 100 mg/m^2^, methotrexate 100 mg/m^2^ with methotrexate 200 mg/m^2^ for 12 h, folic acid 15 mg and actinomycin D 0.5 mg, alternating with cyclophosphamide 600 mg/m^2^ and vincristine 1 mg/m^2^). Prior to chemotherapy, her β-human chorionic gonadotropin (β-HCG) level was 12,162.53 U/L; after two courses of therapy the serum β-HCG level had changed significantly (66,486.37 U/L); and after the third course, β-HCG level had dropped by more than tenfold. The patient’s general condition was not poor. Laboratory tests showed that the leukocyte level had fallen to 2.7–3.2 × 10^9^/L, but all other results (liver and renal functions, urinalysis, serology, microbiology) were about normal. The patient was administered recombinant human granulocyte-colony stimulating factor by hypodermic injection to promote the leukocyte level, resulting in to 8–15 × 10^9^/L. After eight cycles of chemotherapy, the β-HCG level had returned to normal (3.68 U/L) (Fig. 4). The patient was administered ten cycles chemotherapy and was fortunately able to tolerate full-dose chemotherapy.

### Follow-up

After eight weeks, the newborn baby was discharged in good condition, and she is developmentally normal with no cognitive or motor delays. The patient remains disease free based on the β-HCG level and radiology examination for 1 year. The β-HCG level and the results on the CT scans of the brain and lung are within the normal range.

## Discussion

Metastatic choriocarcinoma in a patient at a late stage of pregnancy is extremely rare. It is more likely to be associated with hydatidiform moles, and even then only 2–3% of hydatidiform moles progress to choriocarcinoma [1]. Our patient presented with severe and unremitting headaches, nausea, and vomiting, all typical systems of choriocarcinoma. The CT scans of the brain and lungs were highly suspicious of pulmonary tuberculosis metastasis; however, the preliminary diagnosis was a misdiagnosis. Fortunately, sequential management of the patient was not delayed. We observed a significant rise in the level of β-HCG. A background search of the patient's menstrual and obstetrical history revealed that the last artificial abortion was mild trophoblastic hyperplasia. The patient did not have a follow-up examination of HCG level. It was considered that trophoblastic hyperplasia evolved into choriocarcinoma in the current pregnancy. Pathological examination of the brain hemorrhagic mass supported the diagnosis of choriocarcinoma.

Yang *et al*. [2] reported a case of gestational choriocarcinoma misdiagnosed as pulmonary tuberculosis in a patient who had no abnormal uterine bleeding. When a patient presents with multiple pulmonary nodules, brain multiple mass, or other viscera multiple mass, we must consider choriocarcinoma, whether the patient is pregnant or not, whether or not menstruation has ceased, and whether or not there is irregular vaginal bleeding. The doctor should take note of serum β-HCG levels and obstetrical history.

Mamelak *et al*. reported a case of choriocarcinoma brain metastasis in a patient in her 30th week of pregnancy [3]. The patient was treated with surgical removal of the brain metastasis and a cesarean section. We also performed caesarean section and craniotomy, hematoma clearance, and decompression simultaneously in our patient, and surgically removed a significant mass of brain metastasis. It was noted in our patient that when the brain mass grows progressively, neurological symptoms deteriorate promptly.

Chemotherapy offersa cure to 80% of choriocarcinoma metastases, even those to the lung, brain, or other any body part [4]. One case report showed that a patient with metastatic choriocarcinoma treated with EMACO chemotherapy achieved satisfactory clinical prognosis [5]. Yu *et al*. reported a case of patient with metastatic choriocarcinoma who required combination chemotherapy as EP/EMA (toposide, methotrexate, and dactinomycin) [6]. Mamelak *et al*. reported a case of brain metastatic choriocarcinoma that was treated with brain radiotherapy and chemotherapy within 1 week after surgery [3]. Brain metastatic choriocarcinoma requires multidrug combined therapy, including craniotomy, whole brain radiotherapy, and EP-EMA or EMA-CO chemotherapy [7]. However, the effectiveness of radiotherapy is still debatable. In our case, the patient was very satisfied with her recovery on the EP and EMACO regimens.

We searched PUBMED and Web of Knowledge for studies on intrauterine pregnancy associated with brain metastatic choriocarcinoma. We found that choriocarcinoma brain metastasis presented between 28 and 32 weeks of pregnancy [3, 6, 8, 9]. Lorna *et al*. [8] reported a case of metastatic choriocarcinoma in which the initial CT of the brain was negative for any lesions in the 20th week of intrauterine pregnancy. The patient developed intracranial metastases after the induction of labor at 32 weeks of gestation. The time to development of brain metastasis was unknown.

## Conclusions

Gestational choriocarcinoma in a third trimester pregnancy is rare, but metastatic lesions are numerous. If an intracranial mass or bilateral pulmonary nodules are found in a pregnant woman, especially during the third trimester, metastatic choriocarcinoma should be considered.

## Data Availability

We make readily reproducible materials described in the manuscript.

## Abbreviations

CT: Computerized tomography
EMA: Etoposide, methotrexate, and dactinomycin
EMACO: Etoposide, methotrexate, and dactinomycin alternating with cyclophosphamide and vincristine
EP: Etoposide, cisplatin
β-HCG: β-Human chorionic gonadotropin
SCI: Web of Knowledge
